# Long-Term Outcomes of Occlusal Force Management in Severe Maxillary Canine Regeneration: A Report of Two Cases

**DOI:** 10.7759/cureus.80085

**Published:** 2025-03-05

**Authors:** Yuichiro Ihara, Satoru Morikawa, Taneaki Nakagawa

**Affiliations:** 1 Dentistry and Periodontology, Ihara Dental Clinic, Tokyo, JPN; 2 Dentistry and Oral Surgery, Keio University School of Medicine, Tokyo, JPN

**Keywords:** alveolar bone loss, canine tooth, dental occlusion, enamel matrix derivative, long term outcome, periodontal regeneration

## Abstract

Maxillary canines present unique challenges in periodontal regenerative therapy due to their complex biomechanical roles. However, the long-term effectiveness of combining periodontal regenerative therapy and occlusal force management for severely compromised maxillary canines remains unclear.

This report aims to address these challenges. Two cases of severe periodontal bone loss in maxillary canines, extending to the apical region, were treated with regenerative procedures and occlusal force management. Treatment involved initial periodontal therapy, regenerative therapy using the modified papilla preservation technique with enamel matrix derivative and bone grafts, and distributing occlusal force to both the maxillary canine and premolar while eliminating premature contact. The cases were followed up for eight and five years, respectively. Both cases showed significant improvement, with the probing depth reduced by 3-4 mm and corresponding improvements in clinical attachment levels. Case 1 required two regenerative procedures and prosthetic intervention while Case 2 achieved stability with a single procedure. Long-term follow-up demonstrated stable periodontal tissues and radiographic evidence of bone regeneration. Tooth mobility was reduced from grade II to grade 0 in both cases.

This report suggests that integrating periodontal regeneration with careful occlusal force management can result in long-term stability for severely compromised maxillary canines. These findings may inform treatment protocols for complex cases involving teeth subjected to significant biomechanical stress.

## Introduction

Occlusal forces play a complex and often debated role in the progression of periodontal disease. While excessive occlusal forces alone do not cause alveolar bone resorption, they can exacerbate bone resorptions when combined with periodontal inflammation [1]. Studies suggest that occlusal trauma coexisting with periodontitis may worsen alveolar bone resorption beyond the effects of bacterial factors alone [2,3]. This interaction highlights the need for treatment strategies that address both infection and occlusal forces, particularly for teeth subjected to complex biomechanical stress. Understanding this relationship is crucial for improving long-term outcomes of periodontal therapy, particularly in challenging cases.

Performing periodontal regenerative therapy on maxillary canines presents unique challenges. These teeth bear significant vertical forces and guide lateral mandibular movements, subjecting them to multidirectional stress [4]. Increased tooth mobility after treatment has been reported to be correlated with reduced clinical attachment gain in regenerative procedures. Moreover, teeth with high mobility (Miller classification grade II or higher) frequently require splinting postoperatively [5-7]. Despite routine occlusal adjustment in periodontal treatment [8], clear guidelines for occlusal force management following regenerative therapy remain lacking.

Recent studies have elucidated the molecular mechanisms linking mechanical forces to periodontal health. Gingival mechanical stimulation can trigger interleukin-6 (IL-6) production by epithelial cells, leading to T helper 17 (Th17) cell accumulation and alveolar bone resorption [9]. IL-6, an inflammatory cytokine, promotes Th17 cell differentiation, which in turn produces factors that stimulate bone resorption. These findings establish a direct link between mechanical forces and periodontal destruction at the cellular level, emphasizing the need for precise occlusal force management in regenerative therapy, particularly for teeth such as the maxillary canines, which experience complex biomechanical stress.

Herein, we present two cases of severe periodontal bone loss affecting the maxillary canines, with lesions extending to the root apex. These cases demonstrate the interrelationship between periodontal regenerative therapy and occlusal force management. Our approach integrates regenerative procedures with comprehensive occlusal force management, including pre- and postoperative adjustments, splinting, and minimal crown morphology modification. Occlusal adjustments were performed to eliminate premature contact, aiming to distribute the occlusal force to the maxillary canine and premolar. We aimed to address the severe bone resorption in the maxillary canines, providing insights into long-term outcomes over eight and five years. Unlike previous studies that primarily report short-term results, our extended follow-up period offers a unique perspective on the long-term stability of these interventions. By detailing our strategies and outcomes, we hope to contribute to the development of effective protocols for complex cases involving the maxillary canines.

## Case presentation

Patient consent

Patients in this report presented to Ihara Dental Clinic, Tokyo, and Keio University School of Medicine, Tokyo, Japan, and completed the informed consent process.

Case 1

Severe Periodontal Defect in the Maxillary Canines With Secondary Occlusal Trauma

A 51-year-old, non-smoking woman presented for periodontal therapy. Her dental care history was irregular, consisting of occasional visits for caries treatment and calculus removal but no consistent preventive care. She had no relevant medical history that impacted her periodontitis, including diabetes, autoimmune diseases, and osteoporosis.

Clinical Examination

**Figure 1:**
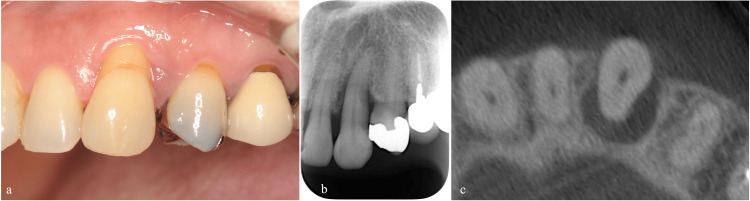
Clinical and radiographic presentation of the left maxillary canine at the initial examination (a) Labial view showing a 3 mm gingival recession. (b) Peri-apical radiography revealing enlarged periodontal ligament space and bone resorption. (c) Cone-beam computed tomography showing significant bone loss on the palatal aspect.

The labial aspect of the left maxillary canine exhibited a 3 mm gingival recession while a 10 mm periodontal pocket was observed on the palatal-distal aspect. Occlusal examination revealed premature contact and grade II mobility (Figure 1a, Table 1). Cone-beam computed tomography and peri-apical radiography confirmed significant bone loss on the palatal aspect, extending to the root apex (Figures 1b, 1c).

**Table 1:** Periodontal parameters at baseline and eight years post-second regenerative therapy PPD: probing pocket depth, CAL: clinical attachment level

		Baseline			8 years		
Tooth number		#23			#23		
		Mesial		Distal	Mesial		Distal
PPD	Labial	3	3	9	3	2	3
	Palatal	6	10	10	3	3	4
CAL	Labial	4	6	10	3	2	3
	Palatal	6	10	10	3	3	4
Mobility		Ⅱ			0		

Diagnosis

According to the 2018 classification of periodontal diseases [10,11], the patient was diagnosed with localized stage III grade C periodontitis with secondary occlusal trauma and type I recession. Cuspid-protected occlusion with premature contact in the occlusal relationship was seen.

Treatment Plan

We planned to perform initial periodontal therapy, which includes an occlusal adjustment on the left maxillary canine to eliminate premature contact. Following that, we evaluated the periodontal pocket and occlusal condition. If periodontal pockets remained with bleeding on probing, periodontal regenerative therapy was planned. The patient consented to this treatment after we explained it. We also planned regenerative therapy with combination therapy based on the patient’s consent.

Initial Therapy

We performed an initial periodontal therapy for a three-month period, which included oral hygiene instruction, scaling, root planing, and occlusal adjustment of the left maxillary canine, which involved eliminating premature contact. The fixation to the adjacent tooth was also not performed because the patient did not have a mastication disorder. Additionally, we provided a stabilization splint for night-time use.

First Regenerative Procedure

**Figure 2:**
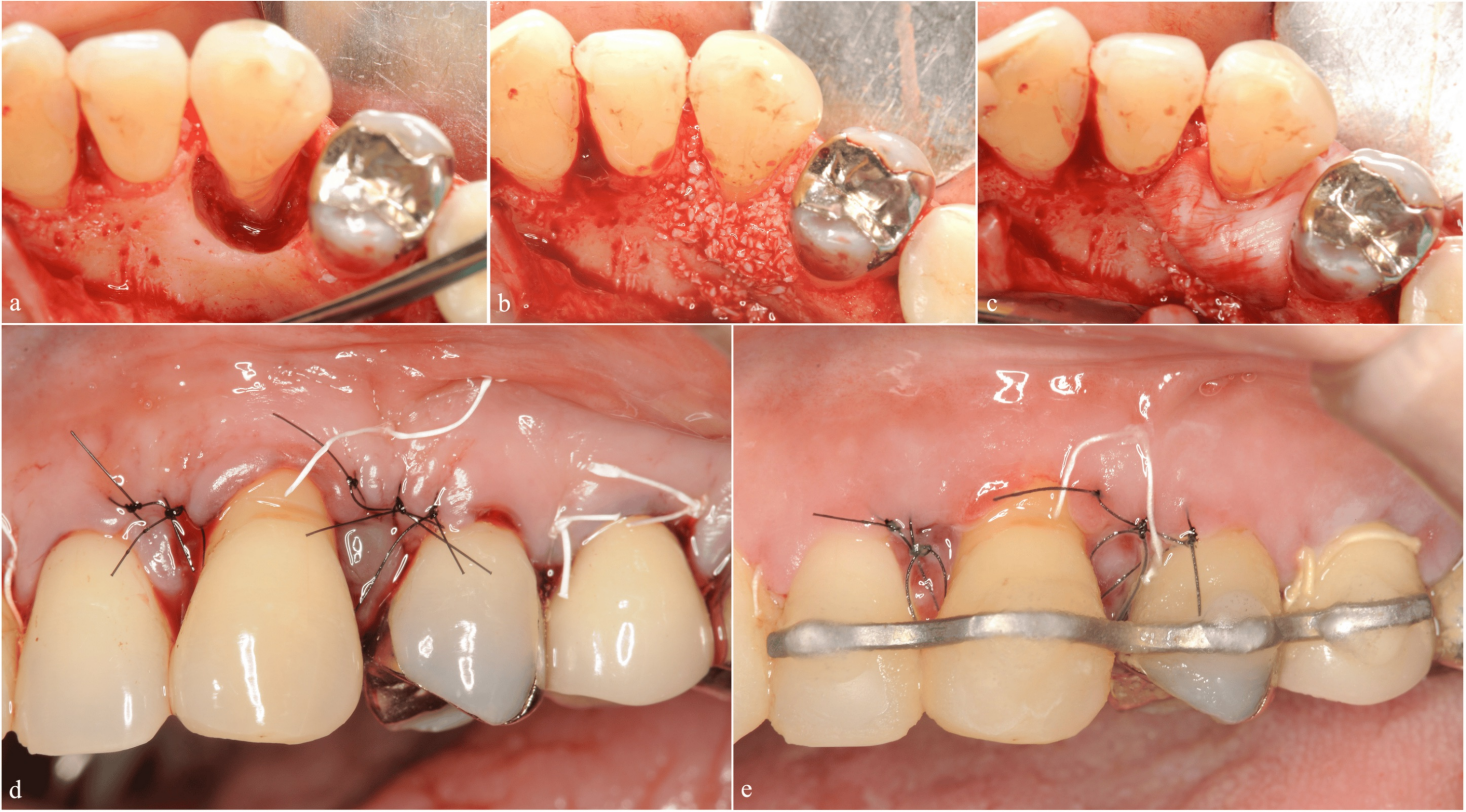
First regenerative therapy using the modified papilla preservation technique (a) Debridement of the root surface and defect. (b) Application of combined enamel matrix derivative and deproteinized bovine bone mineral. (c) Coverage with a resorbable membrane. (d) Flap closure. (e) Primary closure was achieved at 2 weeks postoperatively. Splinting was performed immediately after surgery.

Despite improved tooth mobility (from grade II to grade I), a persistent 10 mm pocket remained. We performed periodontal regenerative therapy using the modified papilla preservation technique (M-PPT) [12]. The procedure included debridement, application of enamel matrix derivative (EMD; Emdogain®, Straumann AG, Basel, Switzerland), and placement of deproteinized bovine bone mineral (DBBM; Bio-Oss®, Gesitlich Pharma AG, Wolhusen, Switzerland). The defect was then covered with a resorbable membrane (Bio-Gide®, Geistlich Pharma AG, Wolhusen, Switzerland), and the flap was repositioned. The tooth was splinted to the adjacent teeth postoperatively (Figures 2a-2e).

Second Regenerative Procedure

**Figure 3:**
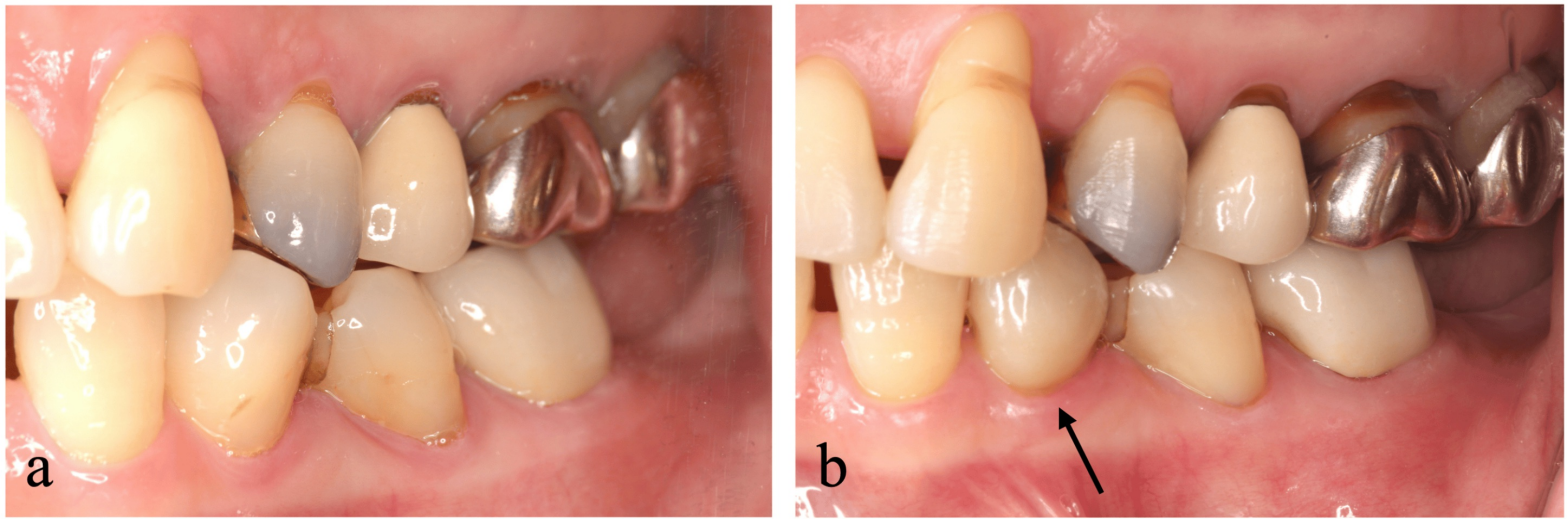
Crown morphology modification to distribute lateral guidance (a) Pre-treatment lateral view showing concentrated forces on the left maxillary canine. (b) Post-treatment view showing crown morphology modification of the left mandibular first premolar to distribute lateral guidance to the left maxillary canine and the left maxillary first premolar.

**Figure 4:**
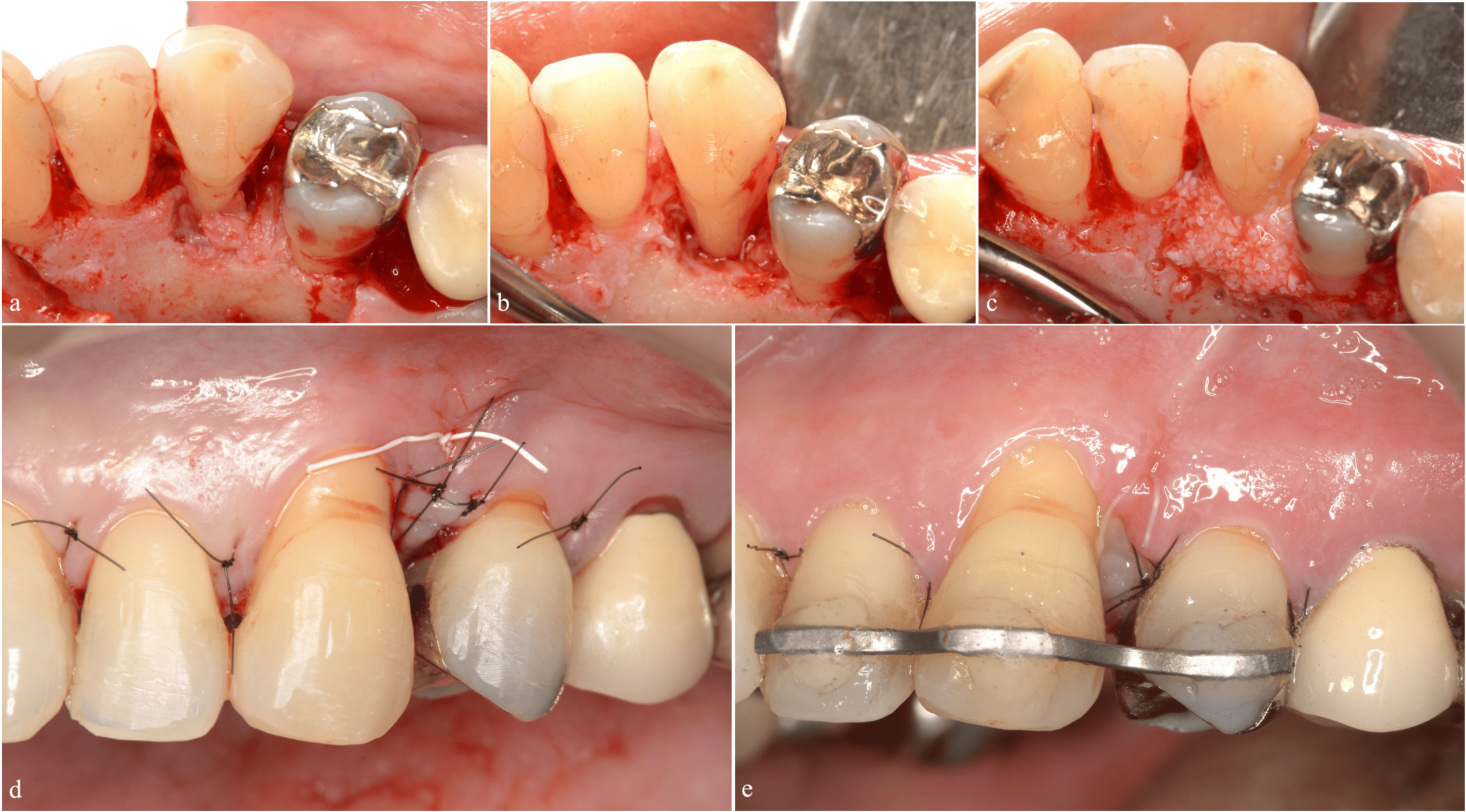
Second regenerative therapy on the palatal aspect (a) Granulation tissue was observed. (b) Debridement was performed. (c) Application of combined enamel matrix derivative and deproteinized bovine bone mineral. (d) Flap repositioning. (e) Primary closure was achieved at two weeks postoperatively. Splinting was performed immediately after surgery.

At the one-year postoperative evaluation, a 5 mm pocket with positive bleeding on probing persisted. Additionally, excessive occlusal force has persisted on the left maxillary canine while guiding mandibular movement to the lateral side. We provided provisional restoration on the left mandibular first premolar to distribute lateral guidance between the left maxillary canine and the left maxillary first premolar (Figures 3a, 3b). Subsequently, we performed a second regenerative procedure on the palatal side using M-PPT, EMD, and DBBM (Figures 4a-4e). The tooth remained splinted to the adjacent teeth for six months postoperatively, as in the initial regenerative therapy.

Postoperative Management

**Figure 5:**
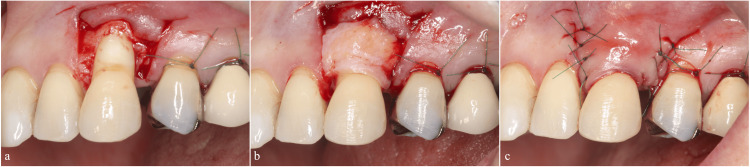
Root coverage using subepithelial connective tissue grafts (a) Trapezoidal flap design and partial-thickness flap elevation. (b) Connective tissue graft placement. (c) Immediate postoperative view.

Occlusal adjustments and splint evaluations were conducted at one-month postoperative follow-up visits after both regenerative surgeries. To address root exposure, we performed a subepithelial connective tissue graft (Figures 5a-5c).

Outcome

**Figure 6:**
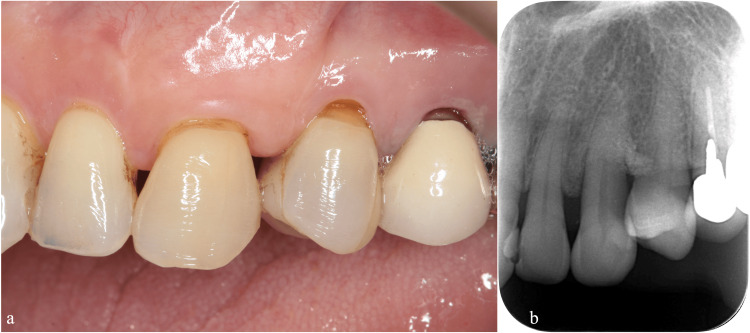
Long-term follow-up at eight years after treatment (a) Labial view showing stable gingival margins with no signs of inflammation. (b) Radiographic view confirming the maintenance of bone level and periodontal health.

At eight years following the second regenerative therapy and more than seven years into supportive periodontal therapy (SPT), the periodontal tissues remained stable without signs of inflammation. Long-term stability was evident (Figures 6a, 6b, Table 1), as demonstrated by reduced probing depths, improved clinical attachment levels, and radiographic evidence of bone fill.

Case 2

Severe Periodontal Defect in the Maxillary Canines With Secondary Occlusal Trauma

A 55-year-old, non-smoking woman presented to our dental office with the chief complaint of gingival swelling. Her dental history included orthodontic treatment and previous periodontal therapy. She had no relevant medical history, including diabetes, autoimmune diseases, and osteoporosis.

Clinical Examination

**Figure 7:**
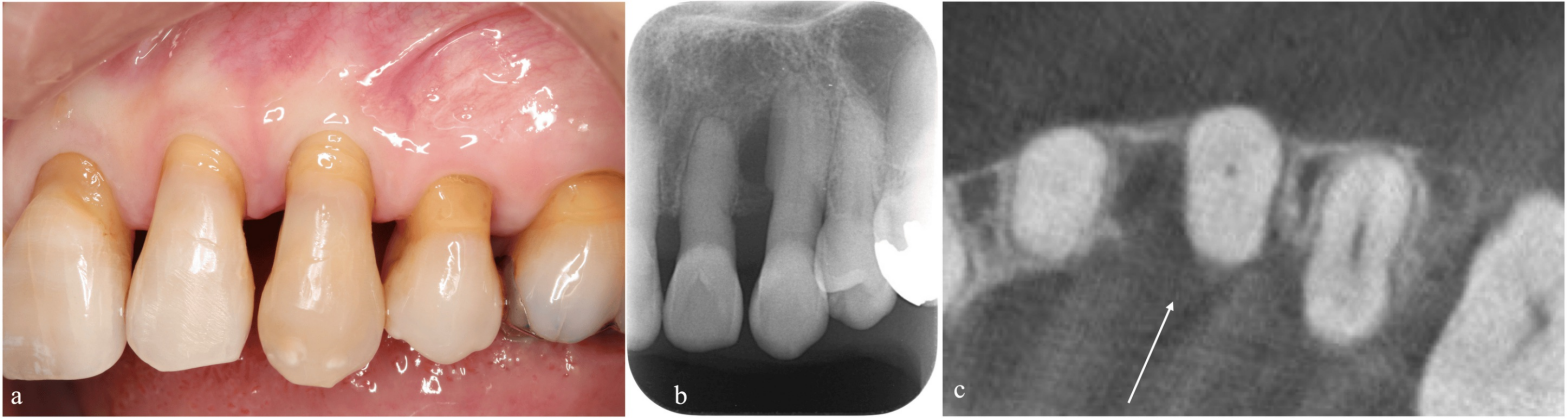
Clinical presentation of left maxillary canine and adjacent teeth at the initial examination (a) Labial view showing gingival recession and interdental papillae loss. (b) Peri-apical radiography showing bone resorption extending near the apex of the left maxillary canine. (c) Cone-beam computed tomography confirming significant bone loss on the palatal aspect.

Our examination revealed gingival recession on the labial aspect of the left maxillary canine and incisors and loss of interdental papillae. We detected a periodontal pocket exceeding 10 mm on the mesiopalatal aspect of the left maxillary canine while 3 mm periodontal pockets were observed on the labial aspects. The tooth exhibited premature contact and grade II mobility (Figure 7a, Table 2). Peri-apical radiography indicated bone resorption extending near the apex of the left maxillary canine (Figures 7b, 7c). Pulp vitality testing revealed a normal response.

**Table 2:** Periodontal parameters at baseline and five years post-surgery PPD: probing pocket depth, CAL: clinical attachment level

		Baseline			5 years		
Tooth number		#23			#23		
		Mesial		Distal	Mesial		Distal
PPD	Labial	10	3	3	3	2	3
	Palatal	10	10	8	3	3	2
CAL	Labial	14	5	8	4	2	4
	Palatal	14	12	10	5	5	3
Mobility		Ⅱ			0		

Diagnosis

According to the 2018 classification of periodontal diseases [10,11], we diagnosed the patient with localized stage III grade C periodontitis with secondary occlusal trauma and type III recession. Cuspid-protected occlusion with premature contact in the occlusal relationship.

Treatment Plan

We planned to perform initial periodontal therapy, which includes an occlusal adjustment on the left maxillary canine to eliminate premature contact and distribute the occlusal force laterally. Following that, we assessed the periodontal pocket and occlusal state. If periodontal pockets remained with bleeding on probing, periodontal regenerative therapy was planned. Informed consent was obtained following the explanation of this treatment by the operator. We planned to adopt combination therapy for periodontal regenerative therapy based on the patient’s consent.

Initial Therapy

We performed a six-month initial periodontal therapy, including oral hygiene instruction, scaling, root planing, and occlusal adjustment. This adjustment involves eliminating premature contact and distributing the occlusal force laterally from the left maxillary canine to both the maxillary canine and second premolar. The fixation to the adjacent tooth was not performed because the patient did not have a mastication disorder and strong pain. A stabilization splint was provided to minimize bruxism-induced occlusal trauma.

Regenerative Procedure

**Figure 8:**
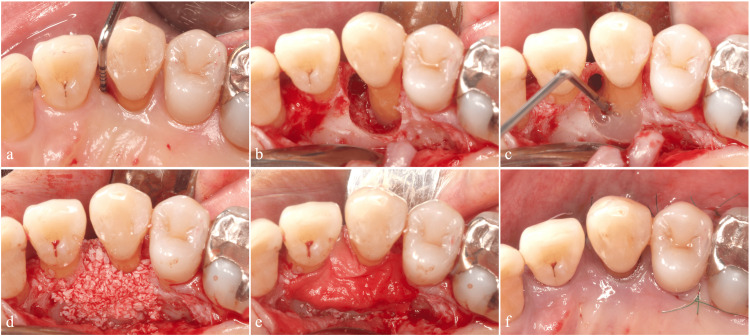
Regenerative therapy using the modified papilla preservation technique (a) Preoperative occlusal view. (b) Root surface debridement. (c) Application of enamel matrix derivative. (d) Placement of deproteinized bovine bone mineral. (e) Coverage with a resorbable membrane. (f) Flap repositioning.

We performed periodontal regenerative therapy using M-PPT. The procedure included root surface debridement, EMD application, DBBM placement, and coverage with a resorbable membrane (Figures 8a-8f).

Postoperative Management

**Figure 9:**
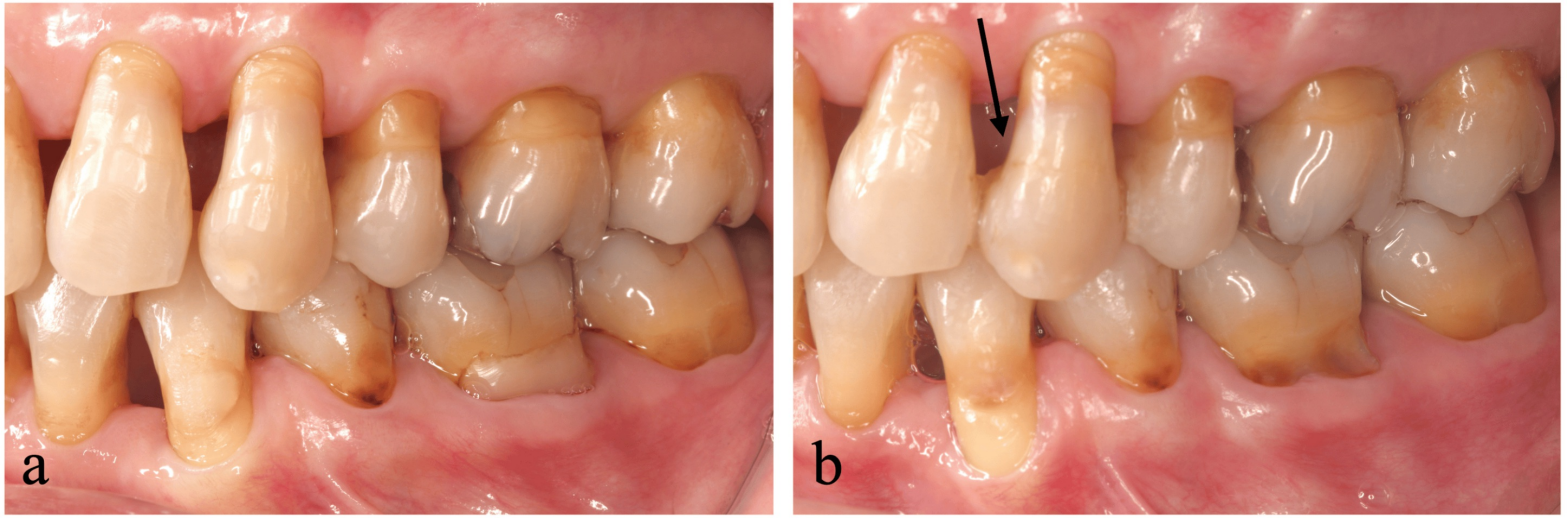
Occlusal adjustment to distribute lateral guidance (a) Preoperative lateral view. (b) Teeth adjacent to the left maxillary canine bonded with 4-META adhesive resin cement. Guidance is established for both the canine and second premolar to prevent excessive occlusal force on the left maxillary canine.

**Figure 10:**
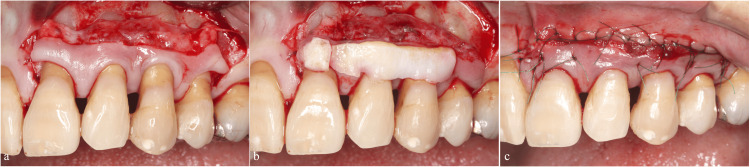
Root coverage using subepithelial connective tissue grafts (a) Full- and partial-layer combination flap was made. (b) Connective tissue graft placement. (c) Postoperative view showing an enhanced gingival contour.

We bonded teeth adjacent to the left maxillary canine using a 4-META adhesive resin cement (Super Bond®, Sun Medical, Moriyama, Japan). This splinting was performed to reduce the occlusal force applied on the left maxillary canine for six months following regenerative surgery. Monthly follow-up visits included an evaluation of the splinting and occlusal adjustments (Figures 9a, 9b). To improve aesthetics and address root exposure, we performed a subepithelial connective tissue graft (Figures 10a-10c).

Outcome

**Figure 11:**
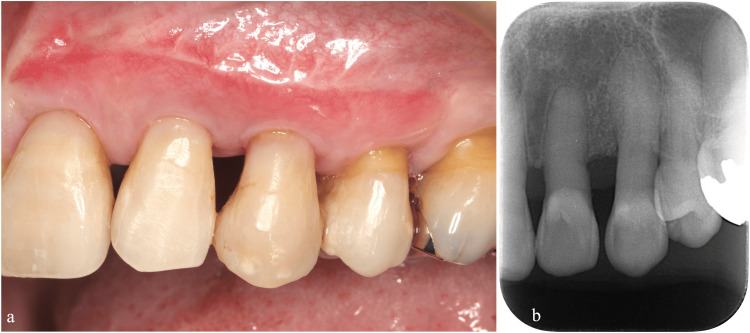
Long-term follow-up at five years post-treatment (a) Intraoral view of stable gingival margins with no signs of inflammation. (b) Radiographic view confirming bone level maintenance.

At 5 years following periodontal regenerative therapy and 4.5 years after SPT, the patient maintained healthy periodontal tissues without signs of recurrent inflammation or disease progression (Figures 11a, 11b, Table 2).

## Discussion

This report on two patients with severe periodontal disease affecting the maxillary canines, characterized by bone resorption extending to the apical region, demonstrated two key findings concerning long-term stability. First, successful periodontal regenerative therapy for severely compromised maxillary canines requires addressing periodontal infection and controlling occlusal forces through pre- and postoperative adjustments, use of occlusal splints, and, when necessary, minimal crown morphology modification. This necessity is due to the unique anatomical position of maxillary canines, which are particularly prone to occlusal trauma given their role in lateral guidance [4]. Second, even in these extreme cases, tooth preservation is achievable through advanced periodontal regenerative therapy incorporating EMD and bone graft materials. These findings, supported by eight- and five-year follow-ups, enhance our understanding of the treatment for severely compromised maxillary canines. They underscore the importance of a comprehensive approach that integrates both biological and biomechanical factors in periodontal regeneration, potentially redefining management strategies for such challenging cases in clinical practice.

The first key finding emphasizes the critical role of occlusal force control in the success of periodontal regenerative therapy for severely compromised maxillary canines. In both cases, extensive bone resorption near the apex of the maxillary canine was likely influenced by a combination of periodontal inflammation and cuspid protected occlusion with premature contact. Furthermore, both cases exhibited similar bone loss on the palatal side. Given that maxillary canine teeth are subjected to traction force on the palatal periodontal ligament during lateral movement, excessive occlusal load may have contributed to inflammation-derived bone resorption. Our treatment strategy, informed by these findings, focused on distributing excessive forces through pre- and postoperative occlusal adjustments, the use of occlusal splints, and, when necessary, minimal crown morphology modification on opposing teeth. This multifaceted approach to force management is further supported by molecular evidence indicating that mechanical stimulation of the gingiva can induce IL-6 production and Th17 cell accumulation, leading to localized inflammation and bone resorption [9]. We considered eliminating premature contact before surgery and distributing occlusal force laterally from the maxillary canine alone to both the maxillary canine and premolar. Clinically, we observed a reduction in tooth mobility from grade II to grade 0 in both cases, indicating the effectiveness of our approach. The long-term stability achieved (eight years in Case 1 despite requiring two procedures and five years in Case 2 with a single intervention) further supports the effectiveness of this comprehensive approach for occlusal force management in periodontal regeneration. However, the feasibility of a second periodontal regenerative therapy may be limited in some clinical settings due to the extended treatment period and patient compliance challenges. These findings underscore the importance of integrating biomechanical considerations into periodontal regenerative therapy, particularly for teeth subjected to significant functional stress, such as the maxillary canines.

Our second significant finding demonstrated that even in cases of severe bone resorption surrounding the root, tooth preservation is achievable through advanced regenerative techniques. In this report, both cases exhibited extensive bone loss with periodontal pockets exceeding 10 mm, yet, long-term stability was achieved using a combined regenerative approach. This outcome can be attributed to several factors, including meticulous plaque control, which is crucial for successful periodontal regenerative therapy, as highlighted by previous studies [13]. In our cases, maintaining excellent oral hygiene allowed for desirable outcomes, even when a second regenerative procedure was necessary in Case 1. The surgical technique employed, specifically the M-PPT, was crucial in preserving the interdental tissues and achieving primary closure, which is essential for optimal wound healing. Furthermore, the combination of EMD and DBBM was strategically selected based on defect morphology [14]. This approach aimed to provide biological signaling for regeneration and space maintenance, which were particularly important for the challenging two-wall defect in Case 2. Despite differences in defect morphology (three-wall defect in Case 1 and two-wall defect in Case 2), the successful outcomes in both cases underscore the potential of a combined regenerative approach for preserving severely compromised teeth. Notably, the need for a second regenerative procedure in Case 1 highlights the importance of ongoing assessment and the willingness to adapt treatment strategies when necessary. Studies on the microvascular response in the periodontal ligament following mucoperiosteal flap surgery highlight the vulnerability of the treated site to occlusal trauma during the healing period [15]. This emphasizes the need for careful load management preoperatively and throughout the healing phase. The success of EMD and DBBM in our cases, even in challenging defect morphologies, reinforces the importance of space maintenance in periodontal wound healing. Furthermore, these cases demonstrate that even when initial regenerative attempts are not fully successful, as in Case 1, reassessment and additional interventions, including crown morphology modifications to distribute occlusal forces laterally, can lead to favorable long-term outcomes. This adaptive approach to treatment underscores the importance of long-term follow-up and willingness to adjust treatment strategies as required.

The present report provides valuable insights into the successful management of severely compromised maxillary canines through periodontal regenerative therapy while avoiding premature contact and excessive occlusal force on the maxillary canine. Periodontal regenerative therapy with occlusal adjustment may prompt the reconsideration of treatment strategies for severe periodontal bone resorption at the maxillary canine. Successful outcomes in such challenging cases depend on multiple interrelated factors, including careful patient selection, appropriate surgical techniques and regenerative materials, and meticulous surgical execution. Additionally, the integration of occlusal force management is paramount, particularly for teeth subjected to significant biomechanical stress, such as the maxillary canines. This includes pre- and postoperative occlusal adjustments and splinting and minimal prosthetic interventions for opposing teeth when necessary. Postoperative stabilization of the treated tooth is crucial to ensure blood clot stability and secure the regenerative scaffold.

## Conclusions

Our cases demonstrate that even severe bone loss extending to the root apex can be successfully managed using advanced regenerative techniques with occlusal management, which includes eliminating premature contact and distributing occlusal force on the maxillary canine. The combination of EMD and bone graft materials, coupled with appropriate surgical techniques, such as M-PPT, resulted in favorable long-term results, as evidenced by our cases with stable outcomes at five and eight years after treatment.

These limitations include the inability to generalize the findings to a broader population, potential bias in case selection, and the absence of a control group for comparison. Future research should focus on developing standardized protocols for occlusal force management in periodontal regenerative therapy and evaluating the long-term outcomes of comprehensive approaches in larger patient cohorts. Ultimately, these cases demonstrate that with meticulous planning, advanced regenerative techniques, and attention to occlusal forces, it is possible to achieve and maintain periodontal health, even in the most challenging scenarios.
